# Anodal tDCS does not enhance the learning of the sequential finger-tapping task by motor imagery practice in healthy older adults

**DOI:** 10.3389/fnagi.2022.1060791

**Published:** 2022-12-09

**Authors:** Angèle Metais, Camille O. Muller, Nawale Boublay, Caroline Breuil, Aymeric Guillot, Sébastien Daligault, Franck Di Rienzo, Christian Collet, Pierre Krolak-Salmon, Arnaud Saimpont

**Affiliations:** ^1^Univ Lyon, Université Claude Bernard Lyon 1, Laboratoire Interuniversitaire de Biologie de la Motricité, LIBM, Villeurbanne, France; ^2^EuroMov Digital Health in Motion, Université Montpellier, IMT Mines Alès, Montpellier, France; ^3^Centre de Recherche Clinique Vieillissement Cerveau - Fragilité, Hospices Civils de Lyon, Lyon, France; ^4^Centre de Recherche Multimodal et Pluridisciplinaire en Imagerie du Vivant (CERMEP), Département de MagnétoEncéphalographie, Bron, France

**Keywords:** transcranial direct current stimulation, motor sequence learning, motor imagery, multisession training, aging, sequential finger tapping task, online learning, offline learning

## Abstract

**Background:**

Motor imagery practice (MIP) and anodal transcranial direct current stimulation (a-tDCS) are innovative methods with independent positive influence on motor sequence learning (MSL) in older adults.

**Objective:**

The present study investigated the effect of MIP combined with a-tDCS over the primary motor cortex (M1) on the learning of a finger tapping sequence of the non-dominant hand in healthy older adults.

**Methods:**

Thirty participants participated in this double-blind sham-controlled study. They performed three MIP sessions, one session per day over three consecutive days and a retention test 1 week after the last training session. During training / MIP, participants had to mentally rehearse an 8-element finger tapping sequence with their left hand, concomitantly to either real (*a-tDCS* group) or sham stimulation (*sham-tDCS* group). Before and after MIP, as well as during the retention test, participants had to physically perform the same sequence as fast and accurately as possible.

**Results:**

Our main results showed that both groups (i) improved their performance during the first two training sessions, reflecting acquisition/on-line performance gains, (ii) stabilized their performance from one training day to another, reflecting off-line consolidation; as well as after 7 days without practice, reflecting retention, (iii) for all stages of MSL, there was no significant difference between the *sham-tDCS* and *a-tDCS* groups.

**Conclusion:**

This study highlights the usefulness of MIP in motor sequence learning for older adults. However, 1.5 mA a-tDCS did not enhance the beneficial effects of MIP, which adds to the inconsistency of results found in tDCS studies. Future work is needed to further explore the best conditions of use of tDCS to improve motor sequence learning with MIP.

## Introduction

Motor sequence learning (MSL) refers to movement sequence retention into procedural memory (Willingham, 1998; Doyon et al., 2018). Learning a new motor sequence can occur either implicitly (i.e., without being aware of the sequence) or explicitly (i.e., with prior knowledge of the motor sequence to be trained). Three phases are usually identified (Doyon and Benali, 2005): (i) initial acquisition, during which performance strongly increases within a single session of practice (online learning), (ii) consolidation, where the motor skill is improved or stabilized in the hours following the acquisition, without further practice (offline learning), and (iii) long-term retention, during which performance slightly increases and is stabilized after several practice sessions (days, weeks, or months of practice). This latter phase involves both online and offline learning processes. Although old people remain able to learn motor skills due to cerebral plasticity, MSL is selectively affected in adults over 65 years (for a review, see King et al., 2013). While performance gains are relatively preserved during the acquisition phase (Shea, 2006; Brown et al., 2009), performance often remains stable or even decreases during the consolidation phase (Spencer et al., 2007; Fogel et al., 2012). The third phase is overall preserved even though additional gains are generally weaker in old adults compared to young people. Age-associated changes in MSL relate to alterations in the neural circuitry, especially in the cortico-striatal network (Censor et al., 2010; Rieckmann et al., 2010; Vien et al., 2016). In parallel, neuronal plasticity is weakened, most especially in the primary motor cortex (M1), thus limiting the efficiency of brain motor functions and learning (Mary et al., 2015; Sawaki et al., 2003, for review see Seidler et al., 2010).

In view of the potential fatigability of the elderly during physical practice (PP), the use of motor imagery (MI), the mental representation of an action without engaging its actual execution (Jeannerod, 1995), may be a relevant alternative or complement to actual execution. Behavioral and neuroimaging studies disclosed that MI shared temporal properties and neural networks with actual execution (Decety et al., 1989; Guillot and Collet, 2005; Collet et al., 2011; Hétu et al., 2013; Grosprêtre et al., 2016). Among them, executed and imagined movements have been shown to activate M1 (Hanakawa et al., 2008; Hétu et al., 2013). As during PP, M1 seems to play a role in all learning phases (Di Rienzo et al., 2016). Sensorimotor areas are also activated during MI in older adults, providing a rationale for its use as a training method in this population (for review, see Saimpont et al., 2013). The temporal similarity between MI and actual execution, as well as MI vividness, are two important dimensions of MI ability. In fine motor skills such as sequential finger tapping tasks (SFTT), these dimensions are preserved with aging (Malouin et al., 2010; Caçola et al., 2013; Saimpont et al., 2015). Practically, a handful of experimental studies provided evidence that MI practice (MIP, i.e., the repetition of imagined movements) contributed to improve motor performance in older adults (for a review, see Marusic and Grosprêtre, 2018). Interestingly, 12 sessions of 6 min of MIP over 6 weeks showed beneficial effects on learning a SFTT (Boraxbekk et al., 2016). The number of finger movements significantly increased, and remained stable 1 week after the end of the practice, reflecting a long-term retention. Additionally, MI vividness has been shown to improve after mental training (Hamel and Lajoie, 2005; Ruffino et al., 2017), reflecting the positive impact of MIP on both physical and mental performance in older adults.

Motor learning has further been shown to be facilitated by transcranial direct current stimulation (tDCS), which consists in delivering a weak current between two surface electrodes (anode and cathode) placed on the scalp. Anodal tDCS (a-tDCS) increases cortical excitability over the stimulated area and induces long-term potentiation (Nitsche and Paulus, 2000; Stagg and Nitsche, 2011). In young adults, a single session of a-tDCS over M1 yielded faster online explicit sequence-learning relative to sham-tDCS (Stagg et al., 2011). Furthermore, several sessions of a-tDCS over M1 exhibited positive offline effects (between-days) and a long-term retention (7 days to 3 months after the last practice session) on a sequential visual isometric pinch task (Reis et al., 2009). Several studies evidenced that a single session of a-tDCS over M1 combined with PP improved upper limb motor functions in healthy older adults (Hummel et al., 2010; Parikh and Cole, 2014; for a review, see Summers et al., 2016). Also, a-tDCS associated with PP promoted learning a complex SFTT, with long lasting effects (offline gains) up to 24 h after the stimulation (Zimerman et al., 2013). Interestingly, combining PP with a-tDCS applied to M1 during five consecutive days of training showed positive online effects on implicit MSL, in the second, third and fourth days (Dumel et al., 2016).

Taken together, MIP and a-tDCS have separately shown their positive impact on MSL in elderly people. Interestingly, a few studies examined the cumulative effects of these methods on the different phases of MSL in young people. Compared to MIP alone, applying a-tDCS over M1 during a single MIP session contributed to improve acquisition of a handwriting task (Foerster et al., 2013), a SFTT (Saimpont et al., 2016), and a postural task (Saruco et al., 2017). Furthermore, three consecutive sessions of a-tDCS, combined with either PP or MIP, improved implicit MSL (Debarnot et al., 2019). In this latter study, MIP benefited from a-tDCS whereas PP did not, with increased online gains during the first acquisition session. There was also a consolidation of performance in the MIP groups only, albeit not enhanced by a-tDCS, reflecting the absence of offline stimulation effects. Finally, the benefits of a-tDCS after three sessions were higher compared to one session, for both practice groups.

To date, no studies investigated the effects of combining MIP and a-tDCS in MSL over multiple training sessions in the elderly. Spurred by the findings in young adults, the present study addressed the effects of three consecutive MIP sessions over 3 days on learning a SFTT in older adults, combined with a-tDCS over M1. We hypothesized that a-tDCS would outperform both online and offline gains elicited as a result of a MIP alone. We secondarily aimed to better understand the MIP processes by exploring the mental performance evolution within and between training sessions. We finally assessed MI ability, i.e., vividness and temporal accuracy, to investigate changes across experimental sessions.

## Materials and methods

### Participants

We included 30 healthy older participants aged from 65 to 80 (mean = 71.53 ± 4.80 years; 16 women) who were recruited *via* local associations. All were right-handed with a personal score higher than 0.5 in the Edinburgh laterality test (Oldfield, 1971). Exclusion criteria were (i) motor disabilities affecting the upper limb (pain, osteoarthritis or arthritis), (ii) performing an activity requiring high dexterity (e.g., playing a musical instrument or video games) more than 5 h a week, (iii) a score lower than 24 at the Mini-Mental State Examination (MMSE; Folstein et al., 1975; Kalafat, 2003), and (iv) a visuospatial span lower than 3 at a digital version of the Corsi block test (Kessels et al., 2000). We also respected the recommended exclusion criteria for tDCS (Thair et al., 2017), i.e., any neurological or alcohol history and/or substance abuse, psychiatric illness, metallic implants, surgical clips or pacemaker, and skin damage. Participants were stratified by gender and pseudo-randomly assigned to an a-tDCS or a sham-tDCS group, according to the type of the stimulation they received during training. We summarize participants’ characteristics in Table 1.

**Table 1:** Summary of groups characteristics.

	a-tDCS	sham-tDCS	Statistics
N	15	15	
Age (years)	71.9 (5.0)	71.1 (4.5)	*p* = 0.66
Gender (M/F)	7/8	7/8	*p* = 1.00
Handedness	0.94 (0.1)	0.94 (0.1)	*p* = 0.94
MMSE	28.5 (1.4)	29.1 (0.8)	*p* = 0.22
Corsi	4.93 (0.6)	4.93 (0.8)	*p* = 1.00

Values indicate means and SD. We performed unpaired Student *t*-tests to compare age, handedness, MMSE and Corsi values and a Chi^2^ test to compare the gender proportions between groups. None of the characteristics was statically different between groups.

The experimental design was approved by the Research Ethics Committee of Lyon Sud-Est IV (CPP number: 16/020). The study was carried out at the Charpennes Geriatric Hospital (Villeurbanne, France). All participants provided their written informed consent and received 120€ as financial compensation for their participation.

### Task and training

**Figure 1:**
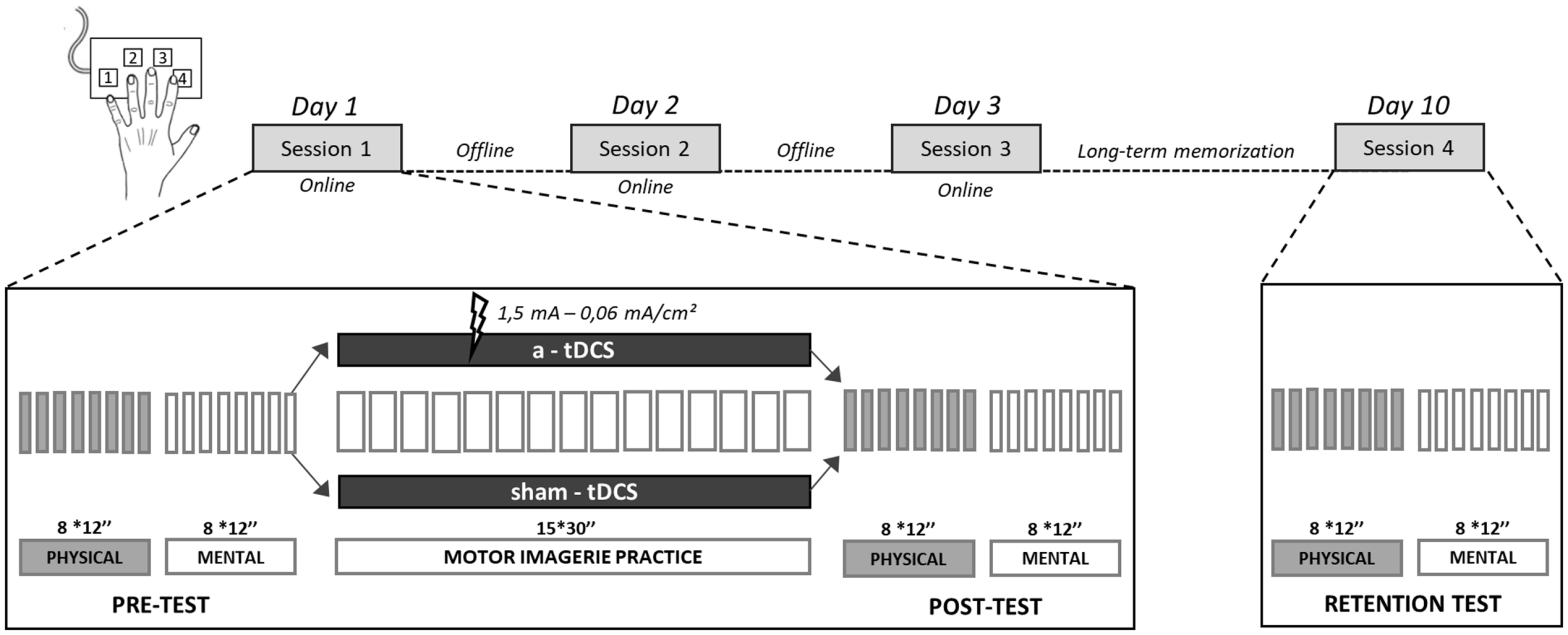
Task and training. The experiment consisted in three consecutive and identical training sessions (sessions 1, 2, and 3) over 3 days followed by a retention test (session 4), 1 week later. The evolution of intra-session performance represented the online learning, and the evolution of inter-session performance represented the offline learning. During each session, participants performed with their left hand an eight-item finger tapping sequence on a gaming keypad: 1-3-4-2-1-4-2-3. Before (pre-test) and after (post-test) MIP, as well as during the retention test, participants had to physically and mentally repeat the sequence, as fast and accurately as possible, during 8 blocks of 12 s (interspaced with 8 s of rest). During MIP, participants mentally rehearsed the sequence, as fast and accurately as possible, during 15 blocks of 30s interspaced with 20s of rest, with either concomitant a-tDCS or sham-tDCS.

Each participant took part in four sessions over a 10-day period. The experimental design consisted in three consecutive training sessions, one session per day, over the course of 3 days, followed by a retention test scheduled 1 week after the last training session (Figure 1). During each session, participants performed a complex SFTT including eight finger movements. They were comfortably seated in a chair in front of a computer, with their forearms resting on the table and their left fingers on a gaming keypad (Razer Nostromo, Razer Inc., United States). The index, middle, ring and little fingers were, respectively, on keys 4, 3, 2, and 1 (Figure 1). The motor sequence required to press each key with the appropriate finger in a predetermined order. The index of the right hand rested on the “enter” key of the computer keyboard, to validate each sequence when performed. We used E-prime software (v1.1 Psychology Software Tools, Inc., United States) to run all training blocks and tests (pre-, post- and retention tests) with automated recording of key presses.

#### Session 1

##### Familiarization

Participants familiarized themselves with the device and the task by explicitly learning a simple eight-item sequence of finger tapping: 1-2-3-4-4-3-2-1. They memorized the sequence by watching a video of the correct sequence performed by a model and shown from a first perceptive. They watched this video as many times as needed to physically perform three consecutive sequences without any error. Once memorized, they repeated the sequence, as fast and accurately as possible, during three blocks of 12 s, first physically, then mentally. They started with the physical then the mental tests to facilitate accurate motor representations (Schuster et al., 2011). Moreover, performing the task physically jute before MI positively influence the estimation of the temporal characteristics of the movements (Saimpont et al., 2021). Blocks were separated from each other by an 8 s-rest period. During the physical test, participants physically performed the sequence. Each key press was recorded, even if the sequence was not completed at the end of the block. During the mental test, they imagined the same sequence, while keeping their hand motionless on the keypad. They were requested to perform first-person MI, by combining visual and kinesthetic/tactile informations? After physically or mentally completing the whole sequence, participants pressed the “enter” key with their right index, then started the next one. If they perceived an error, they pressed the “enter” key to start another sequence without completing the erroneously perceived one. At the end of each block (either physical or mental) a sound signal indicated to passively fix a cross on the computer screen. When the 8 s of rest were over, the cross disappeared and a written information instructed participants to press the “enter” key to start the next block.

##### Memorization of the SFTT

After familiarization, participants learnt the complex eight-item sequence: 1-3-4-2-1-4-2-3. As during familiarization, they watched a video showing the correct sequence as many times as required for them to be able to correctly perform the sequence three times in a row without error.

#### Sessions 1, 2, and 3

##### Pre-test

After the memorization stage in the first session, or directly at the beginning of the second and third sessions, participants performed the pre-test which was divided into a physical part followed by a mental part. The pre-test required the participants to either physically or mentally repeat the complex sequence, as fast and accurately as possible, during 8 blocks of 12 s, interspaced by 8 s of rest.

##### Motor imagery practice

MIP started after the pre-test. Participants mentally repeated the sequence as fast and as accurately as possible during 15 blocks of 30s interspaced by 20s of rest. Participants in the a-tDCS group received a real stimulation while those in the sham-tDCS group received a sham stimulation during MIP. The stimulations features are described in the Transcranial direct current stimulation section.

##### Post-test

Two minutes after MIP, all participants performed both the physical and mental post-tests. Post-tests conditions were comparable to those of the pre-tests.

#### Session 4

##### Retention test

The retention test was performed 1 week after the third training session. Participants started by recalling and physically performing the complex sequence three times successively, without any error, to check for long-term memorization. Then, they performed a physical and mental retention test under the same conditions as during the pre- and post-tests of the three previous sessions.

### Transcranial direct current stimulation

The stimulation was a double-blinded sham-controlled design. We used the Starstim 7 system (Neuroelectrics, Barcelona, Spain) to deliver the a-tDCS. The stimulation system was connected to a computer *via* Bluetooth, and controlled by NIC2 software (Neuroelectrics Instrument Controller, NIC v2.0). To ensure proper electrodes placement, we used an appropriate electrode cap size according to the head size of each participant.

During each MIP session, we applied a-tDCS or sham-tDCS during 13 min. Similarly to previous studies in the field, we choose to stimulate 13 min at 1.5 mA (Goodwill et al., 2013; Puri et al., 2016). The current was applied through two saline-soaked sponge electrodes. The anode (active electrode of 25cm^2^) was centered above the hand region of right M1 (C4 according to the international 10–20 EEG system), corresponding to the left (trained) hand (Saimpont et al., 2016). The cathode (reference electrode of 35 cm^2^) was placed on the supraorbital ipsilateral region (Fp1). We choose a larger cathode than the anode to reduce the negative flow under this electrode (Nitsche et al., 2007). In the a-tDCS group, the current intensity was gradually increased during 30s until 1.5 mA, kept constant during 13 min, and then gradually decreased during 30s until 0 mA (current density = 0.06 mA/cm^2^). Participants of the sham-tDCS group received a sham stimulation, consisting in a gradual current increase during 30s until 1.5 mA, followed by a gradual decrease during 30s until 0 mA. This ramping up/down replicated the same cutaneous sensations at the stimulation site (e.g., itching/tingling sensations) as those experienced during real simulation. This is a common sham stimulation control used in numerous studies (Nitsche et al., 2008). Both participants and the experimenter were blinded to the type of stimulation. In addition, we controlled the participants’ opinion about the nature of the stimulation that they received. At the end of the last session, they were asked if they thought they received a real stimulation by answering “Yes,” “No” or “I do not know.” No differences in the proportion of responses were found (see Supplementary Table S1). Regardless of the stimulation group, participants were told that they could experience sensations associated with stimulation. Participants were requested to report negative adverse effects, from 0 (no effect) to 10 (worse effect), 1 min before and 45 s after the start of the stimulation, by means of a specific questionnaire (Brunoni et al., 2011). If they reported an effect higher than 5, the experimenter had to stop the stimulation. No participants reported a score higher than 5/10 at any time of the evaluation and the great majority of reported effects were closed to 0 (see Supplementary Table S2).

### Motor imagery ability

#### Session 1

We assessed general MI ability during the first session. We used the short version of the Kinesthetic and Visual Imagery Questionnaire (KVIQ-10, Malouin et al., 2007). The KVIQ assesses MI vividness of five simple movements. For each movement and modality of MI (visual and kinesthetic), participants were requested to i) physically perform the movement, ii) imagine the movement and self-rate the vividness of MI with an analogical scale from 1 (no image associated with no kinesthetic sensation) to 5 (image as clear as during the actual movement along with similar perceived sensations).

#### Sessions 1, 2, 3, and 4

During the four sessions, we assessed task-specific MI ability. They self-reported their MI vividness after each mental test (pre-test, post-test, and retention test) using the same visual and kinesthetic scales as those in the KVIQ-10. We also explored the temporal accuracy of MI by comparing the mean number of keypresses (reflecting the duration of movement) mentally and actually performed during the mental and physical tests, respectively (pre-tests, post-tests, retention test). Note that we estimated the number of imagined keypresses by multiplying by eight the number of imagined sequences, as proposed by Freitas et al. (2020) with a sequential footstep task.

### Complementary measures

At the beginning and the end of each session, participants rated their own sleepiness level with the Stanford Sleepiness Scale (Maclean et al., 1992), from 1 (awake) to 8 (sleepy). They also reported the quality of their previous night on a Likert scale from 1 (very bad) to 5 (very good) and reported the number of hours of sleep.

### Data and statistical analysis

We performed statistical analyses using the R free software (version 1.3). We used linear mixed-models with participants as random effect (lme function, nmle package, v3.1-159; Pinherio and Bates, 2021). Visual inspection of the residual plots did not reveal any deviations from homoscedasticity or normality. The statistical significance threshold was α = 5% and we applied the Bonferroni correction for multiple post-hoc testing. We calculated the intended sample size using G*Power (v3.1.9.4) for repeated measures and within-between interaction design. An *a priori* power calculation (*f* = 0.5, α = 5%, 1–β = 0.85) based on the study by Saimpont et al. (2016) which led us to expect a medium effect size resulted in a total sample size of 20 participants. To prevent for probable attrition and/or data losses (around 20%) and as there is a greater variability in performance with age, we decided to increase the number of participants to 30.

#### Motor performance

We assessed motor performance through the number of correct keypresses performed with the appropriate fingers, within a 12 s-time window. This is an index of both speed and accuracy (Freitas et al., 2020). For each participant and physical test, we calculated the mean number of correct keypresses over the eight blocks of each test. We compared these data by including TEST (pre-test1, post-test1, pre-test2, post-test2, pre-test3, post-test3 and retention), GROUP (a-tDCS or sham-tDCS) and their interaction as fixed effects. To better characterize online learning, we further calculated the variation of correct keypresses between the pre and post-tests for each participant and training session. The dependant variable was an increase rate (%) calculated as follows: $\frac{\left(Mean\;Post-testvalue\right)\;-\;\left(Mean\;Pre-testvalue\right)}{\left(Mean\;Pre-testvalue\right)\;}*100.$

We compared the mean increase rates by including SESSION (S1, S2, S3), GROUP (a-tDCS or sham-tDCS) and their interaction as fixed effects.

Then, to better characterize the offline effects, we calculated, the variation of correct keypresses between each training session (i.e between sessions 1 and 2, and between sessions 2 and 3), for each participant, as follows: $\frac{\left(Mean\;Pre-testvalue\right)\;\;-\;\;\left(Mean\;Post-testvalue\right)}{\left(Mean\;Pre-testvalue\right)}*100$

We compared the mean increase rates by including BETWEEN-SESSION (S1-S2, S2-S3), GROUP (a-tDCS or sham-tDCS) and their interaction as fixed effects.

#### Motor imagery practice

To explore MIP performance, we calculated the mean number of imagined keypresses for each participant, block of practice, and training session. As for mental tests, we estimated the number of imagined keypresses by multiplying the number of imagined sequences by eight (the number of items in the sequence). To analyze the evolution of this variable, we included BLOCK (from 1 to 15), SESSION (S1, S2, and S3), GROUP (a-tDCS or sham-tDCS) and their interaction as fixed effects.

#### Motor imagery ability

##### Vividness

For the general MI ability, we calculated the KVIQ scores for each participant, in the visual and kinesthetic modalities, by summing the vividness scores of the five movements. We compared these scores between groups by means of Wilcoxon tests (normality was violated). We also performed a correlation analysis between these KVIQ scores and the averages of the three performance increases of the number of correct keypresses performed physically. For the task-specific MI ability, we calculated the mean vividness score by averaging the visual and kinesthetic scores for each participant and mental test. We then compared vividness of the imagined sequence by entering TEST (pre-test1, post-test1, pre-test2, post-test2, pre-test3, post-test3 and retention test), GROUP (a-tDCS and sham-tDCS) and their interaction as fixed effects. We also tested the correlations between specific vividness scores at post-tests and the increase rates between pre- and post-tests, for each session.

##### Temporal accuracy

To examine whether executed and imagined presses shared temporal similarity, we calculated, the following index:

(*Number of imagined keypresses)/(Number of physical keypresses*) for each participant and test. The closer the index to 1, the better the temporal accuracy of MI. The indices were then compared by including the effects of TEST (pre-test1, post-test1, pre-test2, post-test2, pre-test3, post-test3 and retention test), GROUP (a-tDCS and sham-tDCS) and their interaction as fixed effects. We also tested the correlations between temporal accuracy scores at post-tests and the increase rates between pre- and post-tests, for each session.

#### Complementary measures

To compare the levels of sleepiness between training sessions and groups, we entered SESSION (S1, S2, and S3), MOMENT (Begin, End), GROUP (a-tDCS or sham-tDCS) and their interaction as fixed effects. Then, to compare the number of reported hours of sleep and quality of the night between training sessions and groups, we entered SESSION (S1, S2, and S3), GROUP (a-tDCS or sham-tDCS) and their interaction as fixed effects.

## Results

### Motor performance

**Figure 2:**
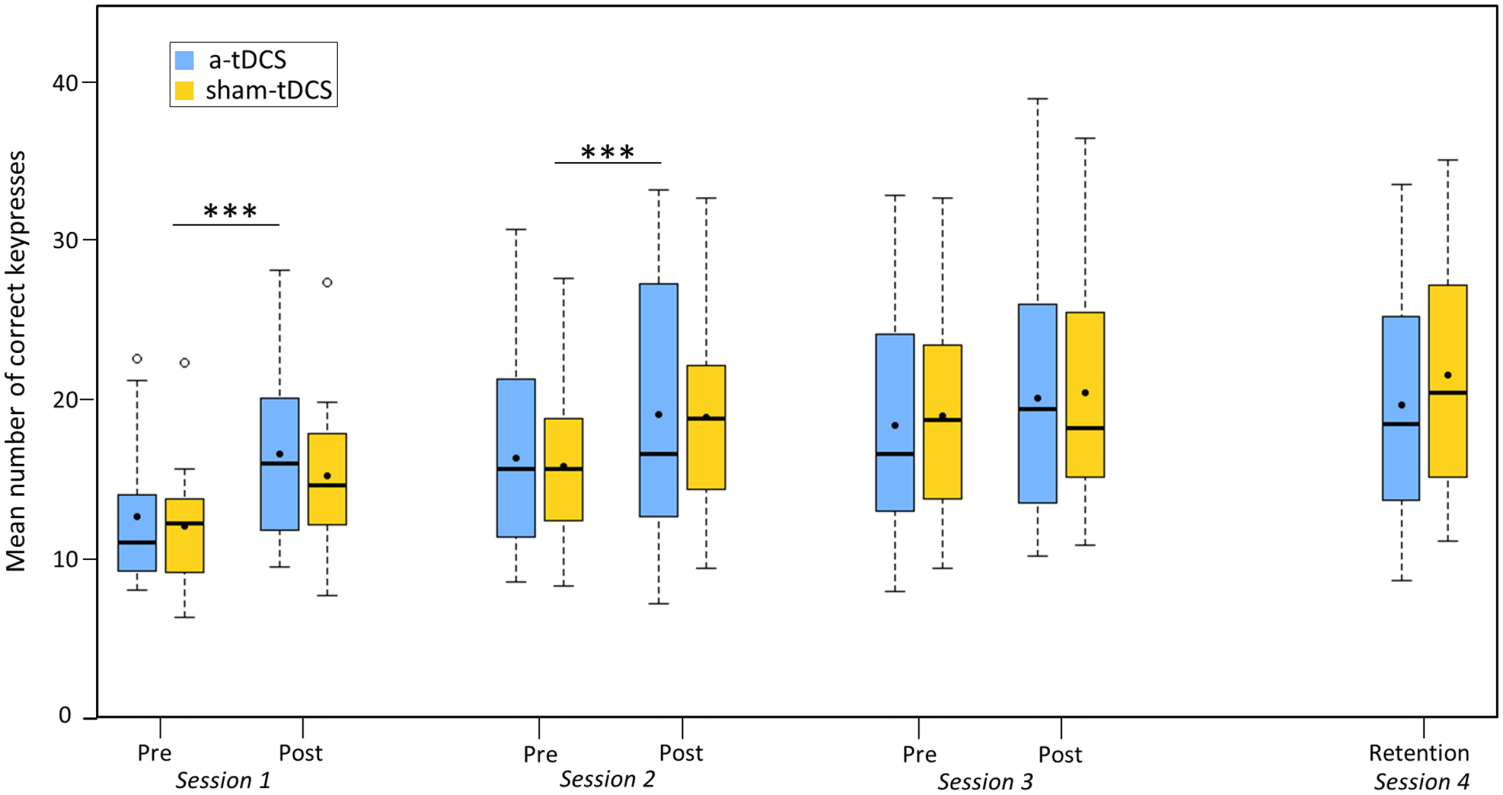
Motor performance. Boxplot of the number of correct keypresses during the different tests (pre-tests, post-tests, and retention test) for the four sessions, the sham-tDCS (yellow curve) and the a-tDCS (blue curve) groups. Dots within each boxplot represent the mean. *** = *p* < 0.001, main effect of TEST.

The analysis of the correct keypresses revealed a TEST effect [χ^2^(6) = 216.44, *p* < 0.001, *η^2^* = 0.56], but no GROUP effect [χ^2^(1) = 0.00, *p* = 0.99], or GROUP*TEST interaction [χ^2^(6) = 6.49, *p* = 0.37]. The number of correct keypresses significantly increased between pre-test 1 and post-test 1 (from 12.35 ± 4.28 to 15.93 ± 5.51, *p* < 0.001), between pre-test 2 and post-test 2 (from 16.11 ± 5.94 to 19.04 ± 7.42, *p* < 0.01), but not between pre-test 3 and post-test 3 (from 18.73 ± 7.13 to 20.31 ± 7.65, *p* = 0.54). No significant difference emerged between post-test 1 and pre-test 2 (*p* = 1.00), and between post-test 2 and pre-test 3 (*p* = 1.00). Finally, there was no significant difference between post-test 3 and retention test (from 20.31 ± 7.65 to 20.70 ± 7.73, *p* = 1.00), as shown by Figure 2).

The analysis of the increase rates of correct keypresses between pre- and post-tests (online learning) revealed a SESSION effect [χ^2^(2) = 19.87, *p* < 0.001, *η^2^* = 0.26], no GROUP effect [χ^2^(1) = 0.14, *p* = 0.70], and no GROUP*SESSION interaction [χ^2^(2) = 0.56, *p* = 0.75]. Post-hoc tests showed that the rate of increase was significantly higher in S1 compared to S2 (from 30.40 ± 21.39 to 17.80 ± 17.20, *p* < 0.05) and S3 (9.86 ± 13.86, p < 0.001). There was no significant difference between S2 and S3 (*p* = 0.28). The analysis of the rates of increase of correct keypresses between post- and pre-tests (offline learning) revealed no BETWEEN-SESSION effect [χ^2^(2) = 0.17, *p* = 0.68], no GROUP effect [χ^2^(2) = 1.56, *p* = 0.21], and no GROUP*BETWEEN-SESSION interaction [χ^2^(2) = 0.35, *p* = 0.55].

### Motor imagery practice

**Figure 3:**
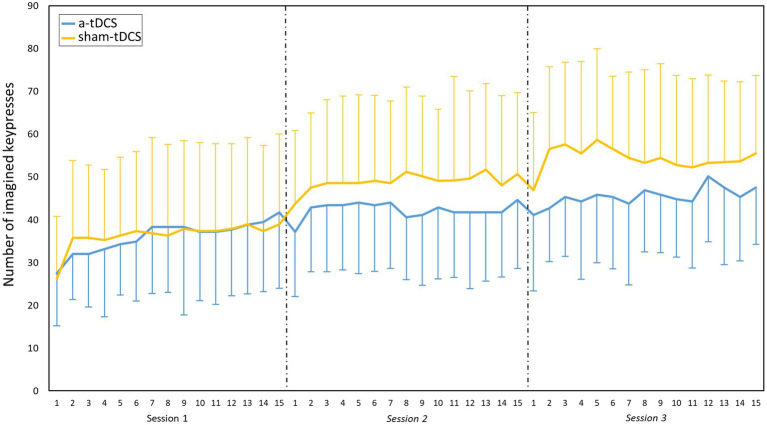
Motor imagery practice. Evolution of the mean (SD) number of imagined keypresses during the three practice sessions. Main effect of BLOCK: significant increase between block 1 and 2 (p < 0.001). Interaction effect of GROUP*SESSION: significant increase between S1 and S2, and between S1 and S3 in both groups. Note that the sham-tDCS group shows higher values than the a-tDCS (p < 0.001), from the second session. NB, the number of imagined keypresses were greater here than in the tests because the duration was different, with 30s-block during training versus 12 s-block during test.

The analysis of the number of imagined keypresses during MIP revealed a SESSION effect [χ^2^(2) = 655.95, *p* < 0.001, *η^2^* = 0.35], a BLOCK effect [χ^2^(14) = 72.38, *p* < 0.001, *η^2^* = 0.06], and a GROUP*SESSION interaction [χ^2^(2) = 46.68, *p* < 0.001, *η^2^* = 0.05], but no other simple or interaction effects. Post-hoc tests on the BLOCK effect revealed that the number of imagined presses significantly increased between block 1 and 2 (from 37.18 ± 16.00 to 43.00 ± 16.69, *p* < 0.001). No other significant changes occurred from block 3 to 15 (Figure 3). Post-hoc tests for the GROUP*SESSION interaction revealed that the number of imagined keypresses significantly increased between S1 and S2, and between S1 and S3 in both groups, but more in the sham-tDCS group (from 36.32 ± 19.01 in S1 to 48.93 ± 18.66 in S2 and to 54.32 ± 19.47 in S3) than in the a-tDCS group (from 36.04 ± 12.45 in S1 to 42.29 ± 11.21 in S2, and to 45.37 ± 14.69 *p* < 0.001 in S3).

### Motor imagery ability

#### Vividness

The analysis of the general MI vividness revealed that the two groups were comparable in terms of KVIQ scores (see Supplementary Table S3). Furthermore, we observed no significant correlation between the general MI vividness scores and the increase rates of motor performance (R^2^ = 0.0064, *p* = 0.66). The analysis of the task-specific MI vividness scores revealed a TEST effect [χ^2^(6) = 13.11, *p* < 0.05, *η^2^* = 0.08] but no GROUP effect [χ^2^(1) = 1.54, *p* = 0.21], and no TEST*GROUP interaction [χ^2^(6) = 8.24, *p* = 0.22]. Post-hoc tests showed that MI vividness significantly increased between pre-test 1 and post-test 3 (from 3.19 ± 0.64 to 3.55 ± 0.66, *p* < 0.05) and remained stable between post-test 3 and retention test (3.56 ± 0.78 and 3.33 ± 0.97, respectively, *p* = 1.00, see Table 2). The correlation analysis revealed no significant correlations between MI vividness at post-tests and increase rates in performance for session 1 (R^2^ = 0.03, *p* = 0.37), session 2 (R^2^ = 0.0016, *p* = 0.81) and session 3 (R^2^ = 0.09, *p* = 0.11).

**Table 2:** Task-specific MI abilities.

		Pre 1	Post 1	Pre 2	Post 2	Pre 3	Post 3	Retention
Vividness	a-tDCS	2.93 ± 0.65	3.03 ± 0.79	3.13 ± 0.64	3.40 ± 0.66	3.17 ± 0.70	3.50 ± 0.71	3.20 ± 0.88
	sham-tDCS	3.43 ± 0.53	3.47 ± 0.58	3.30 ± 0.68	3.33 ± 0.59	3.44 ± 0.65	3.60 ± 0.63	3.47 ± 0.64
Temporal accuracy	a-tDCS	0.88 ± 0.22	1.09 ± 0.48	0.96 ± 0.28	1.00 ± 0.44	0.94 ± 0.30	0.95 ± 0.31	1.00 ± 0.30
	sham-tDCS	1.02 ± 0.38	1.19 ± 0.44	1.25 ± 0.36	1.15 ± 0.42	1.14. ± 0.30	1.15 ± 0.28	1.12 ± 0.25

Vividness: values (mean ± SD) of self-reported MI vividness scores during each mental test from 1 (no images/no kinesthetic perception) to 5 (images as clear as seeing the actual movement/perception as intense as if movement was actually performed). Temporal accuracy: values (mean ± SD) of indices of temporal accuracy (number of imagined keypresses/number of physical keypresses) during each test. The closer the index to 1, the better the temporal accuracy of MI.

#### Temporal accuracy

The analysis of temporal accuracy indices between imagined and executed keypresses revealed a TEST effect [χ^2^(6) = 13.25, *p* < 0.05], no GROUP effect [χ^2^(2) = 2.87, *p* = 0.09], and no GROUP*TEST interaction [χ^2^(6) = 3.46, *p* = 0.75, see Table 2]. Post-hoc tests showed that the temporal accuracy was different only between pre-test 1 (0.95 ± 0.32) and post-test 1 (1.14 ± 0.46, p < 0.05). The correlation analysis revealed no significant correlations between temporal accuracy at post-tests and increase rates in performance for session 1 (R^2^ = 0.11, *p* = 0.07), session 2 (R^2^ = 0.06, *p* = 0.22) and session 3 (R^2^ = 0.02, *p* = 0.40).

### Complementary measures

#### Stanford sleepiness scale

The analysis of the levels of sleepiness during the sessions showed no GROUP effect [χ^2^(1) = 0.11, *p* = 0.73], no SESSION effect [χ^2^(2) = 2.78, *p* = 0.36], no MOMENT effect [χ^2^(1) = 1.66, *p* = 0.19], nor any interaction (see Supplementary Table S4).

#### Sleep

The analysis of the reported hours of sleep revealed a SESSION effect [χ^2^(2) = 6.73, *p* < 0.05, *η^2^* = 0.11], but no GROUP effect [χ^2^(1) = 0.04, *p* = 0.83], and no GROUP*SESSION interaction [χ^2^(2) = 3.26, *p* = 0.20]. Post-hoc tests for the SESSION effect showed that participants reported having slept more the night before the third session (7.36 ± 1.00) compared to the night before the first session (7.12 ± 1.07, p < 0.05, see Supplementary Table S5). The analysis of night quality revealed no SESSION effect [χ^2^(2) = 2.51, *p* = 0.29], no GROUP effect [χ^2^(1) = 0.28, *p* = 0.60], and no SESSION*GROUP interaction [χ^2^(2) = 2.06, *p* = 0.36, see Supplementary Table S5].

## Discussion

The present study investigated for the first time the cumulative effects of three consecutive daily sessions of MIP combined with a-tDCS or sham-tDCS over right M1 on learning a complex SFTT with the left hand, in healthy older adults. This study highlighted the effects of MIP on the different phases of explicit MSL in the elderly. Both groups improved motor performance during the first two training sessions, providing evidence of online learning. In addition, performance was stabilized from one session to another, supporting offline consolidation. Performance further remained stable after 7 days without practice, indicating 1-week retention. However, there was no significant difference in motor performance gains – either online or offline – between participants who received a-tDCS or sham-tDCS. When looking inside MIP processes, both groups improved their mental performance (number of imagined keypresses) with training, and their task-specific MI vividness at the end of the training sessions.

### Motor performance

#### Online gains

Older adults improved motor performance with accumulated online gains during the first two training sessions. The improvement during the first session is in line with previous studies showing that a unique MIP session with older adults led to significant performance gains in SFTT (Caçola et al., 2013). To our knowledge, only Boraxbekk et al. (2016) investigated the effects of several sessions of MIP in learning a SFTT. They demonstrated a positive effect of 12 MIP sessions (over 6 weeks), but without detailing the time-course of online and offline gains for each session. Here, we showed online gains in the first two MIP sessions, with greater gains during the first than the second. This time-course of gains in motor performance is similar to that observed during several PP sessions of a SFTT (Gal et al., 2019) or an implicit sequential task (Dumel et al., 2016) in older adults. In the first session, performance change would mainly be attributable to the memorization of a motor plan needed to learn the SFTT (i.e., the coordination of fingers movement), including the specification of movement parameters (speed, direction, amplitude, i.e., motor programming), allowing to progressively perform faster until speed could no more be improved (the phase of reaching an asymptotic performance). In a computational theoretical framework, internal models allow a predictive mode of motor control, that does not necessary require sensory feedback (Wolpert and Flanagan, 2001; Hardwick et al., 2018). Yet the sensory feedback is not available during MIP. Hence, motor prediction (thus the motor plan and programming as well as the estimation of the future state of the body) could be improved by means of these internal models during that kind of mental training (Gentili et al., 2010; Lebon et al., 2013; Kilteni et al., 2018). In the second session, motor prediction would still be improved with MIP, thus leading to additional gains, while during the third session the prediction could be sufficiently accurate from the start.

#### Offline gains

Older adults did not show off-line gains, since performance was stabilized from one training session to another. Previous studies in young adults have shown that a night of sleep enhanced the consolidation process of explicit MSL after PP, with an overnight improvement of performance (Fischer et al., 2002; Walker et al., 2002, 2003; Korman et al., 2007). Conversely, older adults failed to demonstrate delayed and spontaneous performance improvement after one night post-PP (Korman et al., 2015). This suggests that the sleep-dependent consolidation process is impaired with aging (for review, see King et al., 2013). In fact, decreasing or maintaining performance levels of a SFTT after a 24 h’ offline period is generally observed in older adults (Brown et al., 2009; Gudberg et al., 2015). However, age-related deficit in sleep-dependent consolidation could fade out over several sessions of PP and lead to overall slower rate of learning in older adults (Spencer et al., 2007; Wilson et al., 2012; Gal et al., 2019). Only few studies investigated offline learning processes after MIP in young adults. For a single session of MIP, delayed gains in performance in SFTTs were reported after a night of sleep, but not after a comparable awake time (Debarnot et al., 2009, 2010). However, Ruffino et al. (2021) did not evidence sleep-dependent consolidation in arm pointing task performance. Considering MIP of a motor sequence learnt implicitly over 3 days, Debarnot et al. (2019) showed performance stabilization during the two consolidation periods (between day 1 and day 2, then between day 2 and day 3). To our knowledge, our study is the first to explore offline processes of explicit MSL over several MIP sessions in older adults. We observed performance stabilization overnight between all training sessions, which means that the memory trace elicited by MIP may have been stabilized after sleep without any additional practice. Sleep-dependent performance gains during consolidation would thus depend on task demand (Debarnot et al., 2012), especially in the older population for whom gross motor tasks seem consolidated – after PP – by sleep compared to fine motor tasks (Gudberg et al., 2015). We recently showed sleep-dependent performance gains in young adults, after one session of MIP of a gross motor sequential task involving the whole body (Debarnot et al., 2022). With the age-related alterations in balance, locomotion and sleep, it would be particularly interesting to investigate whether MIP would elicit offline gains in older people in gross motor tasks.

#### One-week retention

We observed that motor performance remained at the same level 1 week after the last training session, without any additional practice. Despite the shorter retention time studied here (7 days), this result is consistent with performance stabilization observed 1 month after ten sessions (spaced over 3 to 4 weeks) of PP of a SFTT (Gal et al., 2019). It is also in line with results by Bonassi et al. (2020) showing that, after four sessions of either MIP or PP of sequential opposition finger movements, the number of correct sequences was maintained 10 days after the end of training. Our result fits well with Doyon’s model describing a long-term retention of motor skills with prolonged practice (Doyon and Benali, 2005; Doyon et al., 2018) and adds evidence in favor of the use of MIP for MSL in healthy older adults.

### Anodal tDCS

In contrast to our hypotheses, several sessions of 1.5 mA a-tDCS associated with MIP did not improve online and offline performances. Actually, previous results on tDCS and motor learning remain divergent. In young adults, a 2 mA a-tDCS over right M1 during a single 13 min-session of MIP enhanced explicit online learning (Foerster et al., 2013; Saimpont et al., 2016). Moreover, several sessions of MIP combined with the same stimulation features (over right M1, 2 mA, 13 min) improved implicit MSL (Debarnot et al., 2019). In older adults, a 1 mA a-tDCS over left M1 during 20 min, simultaneously delivered with one session of PP of a SFTT (Zimerman et al., 2013) or a manual dexterity task (Hummel et al., 2010), facilitated the acquisition and consolidation phases (for review see, Summers et al., 2016). Interestingly, Dumel et al. (2018) investigated the combination of a 2 mA a-tDCS over left M1 during five consecutive sessions of PP of a serial reaction time task in older adults. They found that the benefits of a-tDCS were accumulated during sessions, thus promoting the utility of multisession of a-tDCS design in combination with motor training in the elderly.

However, other studies did not show any impact of a-tDCS on MSL, either by MIP or PP, in young and older adults. For example, no impact of a-tDCS (left M1, 2 mA, 15 min) combined with one MIP session of a finger “Go / NoGo” task was observed on the acquisition and consolidation phases in young participants (Sobierajewicz et al., 2019). In young adults, a-tDCS (over left M1, 2 mA, 20 min) combined with several sessions of PP of a bimanual coordination task did not impact these learning phases (Vancleef et al., 2016). Close to our task, Raw et al. (2016) did not report any evidence of a-tDCS effect (over left M1, 1,5 mA, 30 min) on explicit MSL by PP in different groups of age. In older adults, Greeley et al. (2022) did not show any advantage of two sessions of PP of a discrete sequence production task combined with a-tDCS (over left M1, 2 mA, 20 min) on MSL, compared to sham-tDCS. Puri et al. (2021) even showed that a-tDCS applied over right M1 (1.5 mA, 25 min) during a serial reaction time task altered subsequent performance, when assessed after a 24 h consolidation phase.

Overall, this pattern of divergent results shows that a-tDCS does not elicit systematic positive effects on learning, and highlights the substantial heterogeneity of its effects. In our study, the lack of a-tDCS effect is not likely related to our parameters of stimulation as they were comparable to those of most studies, although we choose a larger cathode (35cm^2^) rather than the most often used (25cm^2^), to reduce the negative flow under this electrode (Nitsche et al., 2007). Nevertheless, we stimulated right M1 as we expected a great room for improvement with the non-dominant left hand in the SFTT, while most research on MSL in older adults placed the anode over left M1 with participants performing the task with their dominant right hand (for review, see Buch et al., 2017). In fact, it has been suggested that the left hemisphere was dominant in controlling motor skills (Grafton et al., 2002; Serrien et al., 2006; Mutha et al., 2012). Moreover, the effects of a-tDCS differed according to the hemispheres, with more pronounced responsiveness in left than right M1 (Schambra et al., 2011). Hence, targeting right M1 might not necessarily be the most effective way for stimulating the aging population.

In addition to M1, motor-related areas, e.g., the premotor cortex and the supplementary motor area, are active during MI (Hanakawa et al., 2008; Guillot et al., 2014).The supplementary motor area is highly activated in the motor-network during MI and plays an important role in information integration (Wang et al., 2019). Furthermore, as aging may affect the effective connectivity between the supplementary motor area and M1, the stimulation intensity (1.5 mA) was possibly too low to promote cortical plasticity during MIP. In this line, higher t-DCS intensity (i.e., 2.3 mA) over left M1 was required in older adults to achieve the same current distribution as in young adults with 2 mA (Indahlastari et al., 2020). Also, Farnad et al. (2021) demonstrated that the cortical excitability of M1 increased by enhancing the intensity of a-tDCS protocol (1 vs. 3 mA) in a 66–80 age group, with long lasting effects 60 and 120 min after the end of the highest stimulation (i.e., 3 mA-20 min, 3 mA-30 min). In addition, individual anatomical factors (skull thickness and composition) would account for up to 50% of the spatial variation of the electric field (Miranda et al., 2006; Opitz et al., 2015) and may also partly explain the lack of a-tDCS effect in our experiment. The moment of stimulation delivery could also impact the expected effect. Several studies investigated the impact of a-tDCS during the consolidation phase of SFTT learning in elderly people (King et al., 2017; Rumpf et al., 2017). The authors showed that stimulating left M1 just after motor acquisition (thus at the beginning of the consolidation) improved the performance compared to a sham stimulation. These results are consistent with neuroimaging studies on activity-dependent plasticity occurring with physical learning of movements, which demonstrated different spatial and temporal patterns of brain activation (Halsband and Lange, 2006; Dayan and Cohen, 2011; King et al., 2013; Doyon et al., 2018). Although M1 seems to play a role in all learning phases, some studies have reported that M1 activity may not change during the acquisition phase (Jenkins et al., 1994; Karni et al., 1998) and increase during the consolidation and retention phases (Karni et al., 1998; Penhune and Doyon, 2002; Penhune and Steele, 2012). It was also shown that M1 was particularly activated a few minutes after skill acquisition, generating an early boost consolidation (Hotermans et al., 2008). Hence, with PP, stimulating at the time of consolidation seems relevant in view of the temporal activation of M1. It is generally admitted that similar brain plasticity and notably M1 pattern activation occurs during motor learning by PP and by MIP, although to a weaker level for MIP (see Di Rienzo et al., 2016 for a review). Interestingly, M1 was involved in the early boost of performance induced after MI training (Debarnot et al., 2011). We should thus further explore whether the effects of a-tDCS depend on the timing with which it is applied relatively to MIP. Post-training stimulation seems a promising way to overcome consolidation deficits in the elderly population.

### Motor imagery practice

#### Online gains

We explored how online gains could occur after MIP by examining the evolution of the mental performance during MIP. Interestingly, for all sessions and groups, the number of imagined keypresses significantly increased between the first two blocks of MIP, then remained relatively stable over the following 13 blocks, suggesting an intra-session mental performance ceiling. Gentili et al. (2010) found comparable learning curves for PP or MIP in learning a pointing sequential task. Briefly, they showed that after an average of 20 trials (i.e., after approximatively 2 min of practice) of an 11-movements sequence, performance became asymptotic for both types of practice. In the present study, the asymptote was observed at the end of the 2^nd^ block (out of 15) and was probably due to previous mental task practice. Indeed, our participants were already involved in mental repetitions of SFTT during the mental pre-test (i.e., eight blocks of 12 s) before MIP. These results emphasize that the state estimation during MIP is solid and accurate (Gentili et al., 2010).

#### Offline gains

The number of imagined keypresses increased between each session, demonstrating offline gains. In other words, although the mental performance reached an asymptote at the second block during each session, it started from a higher level when starting the next session. The motor prediction would be more accurate day after day because the motor system would refine motor commands during the previous physical blocks (i.e., the post-test of the previous day and the pretest of the actual day). Indeed, physical repetition updates the estimation of the sensory consequences of the finger movements with an actualization of visual and kinesthetic information (Di Rienzo et al., 2016). If both groups actually increased their number of imagined key-presses between sessions 1–2 and 1–3, the sham-tDCS group increased performance in a greater extend, suggesting that a-tDCS could impact MI duration. This remains a working hypothesis awaiting further experimental investigation.

### Task specific MI ability

This study also explored MI ability in the elderly and its evolution through MIP. First, according to MI vividness, either the KVIQ scores or the reported vividness scores were, in average, higher than 3. In line with previous studies, this shows that older adults were able to generate and manipulate accurate mental images associated with appropriate movement sensations (Malouin et al., 2010; Saimpont et al., 2013, 2015). However, their general MI ability was not a dependable predictor of performance improvement for this specific finger tapping task. The task specific vividness scores increased between the first and last training session, suggesting that MI vividness also improved after intensive MIP in the elderly, as already observed in young, older and athletic populations (Rodgers et al., 1991; Malouin et al., 2010; Caçola et al., 2013; Saimpont et al., 2015; Ruffino et al., 2017). However, as shown in previous studies using subjective evaluations of MI vividness (Guillot et al., 2010; Ruffino et al., 2017), we did not find any correlation between the vividness scores and the performance enhancements observed after MIP. Even if they had low self-reported scores, participants could increase their performance. This provides prospects for the integration of MI in patients with poor MI ability (Malouin et al., 2013). Secondly, the temporal accuracy changed only during the first session, suggesting that at the beginning of the mental training, participants were slower mentally, probably due to the novelty effects of the training. According to the principle of temporal equivalence, the average index between the number of executed and imagined presses keypresses was 1.06, very close to 1. Thus, participants retained the motor sequence temporal features during MI. In fact, older adults may show temporal similarity between MI and actual execution, although with large interindividual differences (Saimpont et al., 2015). Moreover, subjects with good temporal similarities did not necessarily show the best performance increases, adding to the debate of how interindividual differences in MI abilities influence motor performance improvement (Saimpont et al., 2015; Ruffino et al., 2017).

### Limitations

One limit relates to performance gains after MIP which may partly be explained by the weak amount of PP performed during the physical pre-and post-tests (11% of the total practice). Nevertheless, it is unlikely that performance improvement would come from PP only as the effect size of training was large (*η^2^* = 0.56). The effectiveness of MIP has now been widely demonstrated in the elderly (for review, see Marusic and Grosprêtre, 2018). It would be interesting to test whether adding PP to MIP could cause larger performance gains (Saimpont et al., 2021). Knowing that many factors affect tDCS responsiveness in aging brain structures (Fujiyama et al., 2014), including groups of young participants could also have facilitated results analysis. The age-related neurophysiological changes influencing the responsiveness to tDCS and their relation with MIP need to be further studied to find the best combination of tDCS and MIP in this population. In addition, the effects of various tDCS dosage (higher than 1.5 mA) deserve to be explored to better understand the complex dose–response relationship and to control for possible confounding factors.

Finally, sleep (quality and quantity) was assessed with a participative questionnaire. Yet, aging changes the architecture of sleep which, in turn, reduces the capacity of MSL consolidation (King et al., 2013; Fogel et al., 2017). As inter-individual differences in sleep quality/quantity are decisive (for review, see Ohayon et al., 2004), controlling these parameters more objectively would be of great interest, given the impact of sleep on memory consolidation after MIP (Debarnot et al., 2009; Bonassi et al., 2020).

## Conclusion

Three consecutive daily sessions of MIP improved performance of a SFTT, mainly through online-effects during the first two training sessions. We also showed a stabilization of performance among MIP sessions (off-line consolidation) and a long-term retention of performance 1 week after practice. However, a-tDCS did not enhance the beneficial effects of MIP. Understanding the interaction between a-tDCS and MSL may have important implications for developing rehabilitation research and clinical applications. Future work is needed to further explore the optimal conditions of combining a-tDCS (where/how/when) with MIP in the elderly. Finally, the SFTT-specific MI ability increased with training, emphasizing that the elderly may benefit from MIP to improve personal MI ability.

## Data availability statement

The raw data supporting the conclusions of this article will be made available by the authors, without undue reservation.

## Ethics statement

The studies involving human participants were reviewed and approved by Research Ethics Committee of Lyon Sud-Est IV (CPP number: 16/020). The patients/participants provided their written informed consent to participate in this study.

## Author contributions

NB, AG, CC, PK-S, and AS have made substantial contributions to the conception of the study. CM and CB to the data acquisition. AM, CM, SD, FR, and AS to the data analysis. AM, CC, and AS to the interpretation of data and have drafted the work. All authors contributed to the article and approved the submitted version.

## Funding

This study was supported by the Hospices Civils de Lyon (HCL).

## Conflict of interest

The authors declare that the research was conducted in the absence of any commercial or financial relationships that could be construed as a potential conflict of interest.

## Publisher’s note

All claims expressed in this article are solely those of the authors and do not necessarily represent those of their affiliated organizations, or those of the publisher, the editors and the reviewers. Any product that may be evaluated in this article, or claim that may be made by its manufacturer, is not guaranteed or endorsed by the publisher.
