# Supplementary material for: Anodal tDCS does not enhance the learning of the sequential finger-tapping task by motor imagery practice in healthy older adults
Source: Front Aging Neurosci. 2022 Dec 9;14:1060791. doi: 10.3389/fnagi.2022.1060791 (PMC9780548; doi:10.3389/fnagi.2022.1060791)
Supplement: Supplementary file 1 [file Data_Sheet_1.docx]

Supplementary Material

**Supplementary Tables**

| **TABLE S1 \|** tDCS Blinding. | | | | |
| --- | --- | --- | --- | --- |
|  | **Yes** | **No** | **I don’t know** | Statistics |
| a-tDCS | 5 | 3 | 7 | χ2 (2) = 0.61; p=0.74 |
| sham-tDCS | 7 | 2 | 6 |  |
| Number of participants who thought they received a-tDCS (“Yes”), sham-tDCS (“No”) or don’t know what kind of stimulation they received (“I don’t know”) for each group. To assess blinding regarding the nature of the stimulation, we performed a Chi² test comparing the proportions of the different answers “Yes”, “No” and “I don’t know”. The analysis did not show any significant difference between the groups. | | | | |

| **TABLE S2 \|** Adverse effect during all sessions. | | | | | | |
| --- | --- | --- | --- | --- | --- | --- |
|  | Headache | Neck pain | Scalp pain | Burning | Itching/Tingling | Concentration |
| a-tDCS | 0.04 ± 0.17 | 0.11 ± 0.30 | 0.00 ± 0.00 | 0.02 ± 0.09 | 0.93 ± 1.90 | 0.06 ± 0.18 |
| sham-tDCS | 0.00 ± 0.00 | 0.06 ± 0.11 | 0.00 ± 0.00 | 0.00 ± 0.00 | 0.72 ± 1.72 | 0.00 ± 0.00 |
| Side-effect ratings (mean ± SD) of the participants averaged over the six testing moments (pre and post stimulation, in the three sessions). The presence and intensity of side-effects were rated on a numerical scale ranging from 0 (no sensations) to 10 (extremely strong sensations). | | | | | | |

| **TABLE S3 \|** General MI ability. | | | |
| --- | --- | --- | --- |
|  | **a-tDCS** | **sham-tDCS** | Statistics |
| KVIQ-10 Visual | 17.5 ± 4.0 | 19.3 ± 2.4 | w(29) = 90; p=0.33 |
| KVIQ-10 Kinesthetic | 15.5 ± 4.1 | 14.7 ± 4.9 | w(29)= 132.5; p=0.40 |
| KVIQ-10 scores (mean ± SD) in the two groups. *Abbreviations*: KVIQ-10, short version of the Kinesthetic and Visual Imagery Questionnaire. The KVIQ scores was calculated by summing the scores for the five movements. The analysis did not reveal any significant difference between groups. | | | |

| **TABLE S4 \|** Stanford Sleepiness Scale**.** | | | | | | |
| --- | --- | --- | --- | --- | --- | --- |
|  | **Before** | | | **After** | | |
|  | **Session 1** | **Session 2** | **Session 3** | **Session 1** | **Session 2** | **Session 3** |
| a-tDCS | 1.93 ± 1.03 | 2.00 ± 1.13 | 2.07 ± 0.96 | 2.33 ± 1.18 | 2.00 ± 1.13 | 2.13 ± 1.25 |
| sham-tDCS | 2.47 ± 0.92 | 2.13 ± 0.92 | 1.87 ± 0.64 | 2.20 ± 1.08 | 2.27 ± 0.96 | 2.20 ± 1.01 |
| Levels of sleepiness (mean ± SD), from 1 (awake) to 8 (sleepy), before and after the three training sessions, in the two stimulation groups. The analysis did not show any significant difference between groups, moment and session. | | | | | | |

| **TABLE S5 \|** Sleep Measures. | | | | | | |
| --- | --- | --- | --- | --- | --- | --- |
|  | **Self-reported sleep time** | | | **Quality of the night** | | |
|  | **Session 1** | **Session 2** | **Session 3** | **Session 1** | **Session 2** | **Session 3** |
| a-tDCS | 6.98 ± 0.91 | 7.50 ± 0.90 | 7.43 ± 0.66 | 3.87 ± 0.52 | 4.00 ± 0.85 | 3.60 ± 0.63 |
| sham-tDCS | 7.25 ± 1.23 | 7.23 ± 1.10 | 7.62 ± 0.99 | 3.87 ± 0.64 | 3.67 ± 0.72 | 3.73 ± 0.59 |
| Value (mean ±SD) of self-reported sleep times and indexes of quality of the night for the different training sessions in the two stimulation groups. *Self-reported sleep time*: There was a session effect between Session 1 (7.12 ± 1.07) and Session 3 (7.53 ± 0.82; p=0.05). *Quality of the night*: None significant differences were found between groups and sessions. | | | | | | |
